# ‘Pretty devastating’: exploring horse owner and veterinarian lived experiences of the equine Hendra virus

**DOI:** 10.3389/fvets.2025.1661615

**Published:** 2025-11-21

**Authors:** Jennifer White, Kirrilly Thompson, Debra van den Berg, Genevieve O’Neill, Diana H. Mendez, Joanne Talwar, Chris Degeling, Rebecca Forsythe, David N. Durrheim

**Affiliations:** 1 College of Health Medicine and Wellbeing, The University of Newcastle, Callaghan, NSW, Australia; 2 Hunter New England Local Health District, New Lambton, NSW, Australia; 3 Flinders Health and Medical Research Institute, Flinders University, Adelaide, SA, Australia; 4 Mid North Coast Local Health District, Lismore, NSW, Australia; 5 Graduate Research School, Division Research, James Cook University, Townsville City, QLD, Australia; 6 School of Social Sciences, University of Wollongong, Wollongong, NSW, Australia; 7 New South Wales Department of Primary Industries and Regional Development, Orange, NSW, Australia

**Keywords:** Hendra virus, horse, public health, qualitative, one health, veterinarian, wellbeing

## Abstract

**Introduction:**

With more than 60% of emerging infectious diseases being zoonotic, we apply a One Health lens that connects human, animal, and environmental dimensions of the response to a Hendra virus (HeV) event. One Health promotes collaboration among health professionals, veterinarians, environmental scientists, and policymakers to strengthen health infrastructure and improve responses to complex health threats. HeV is an uncommon high-consequence and potentially fatal zoonotic disease endemic to parts of Australia. Previous research has largely focused on the uptake of preparedness measures by veterinarians and horse owners and less is known about the emotional and experiential factors that may influence their behaviours.

**Methods:**

This study applied Interpretative Phenomenological Analysis to examine how horse owners and veterinarians experience, interpret, and act on HeV risk within a One Health frame of reference. Our aim was to characterise lived experiences at the human–animal–system interface and identify actionable, cross-sector recommendations. We conducted semi-structured interviews with eight horse owners and five veterinarians in a regional area of northern New South Wales, Australia.

**Results:**

Results identified four superordinate themes from the horse owners and two overarching themes from veterinarians. Findings highlight the emotional complexity of recognising and responding to HeV, including grief responses, and implications for future public health strategies. The need for comprehensive support structures underscored by public health liaison with a trusted general practitioner and dedicated access to mental health practitioners experienced in emergency and crisis contexts emerged as an important finding. A need for clear guidance for managing uncertain or deteriorating equine health was identified.

**Conclusion:**

These findings demonstrate how qualitative social science, applied within a One Health framework, can inform targeted messaging, policy considerations and cross-sector responses to emerging zoonoses, including HeV.

## Introduction

**Figure 1:**
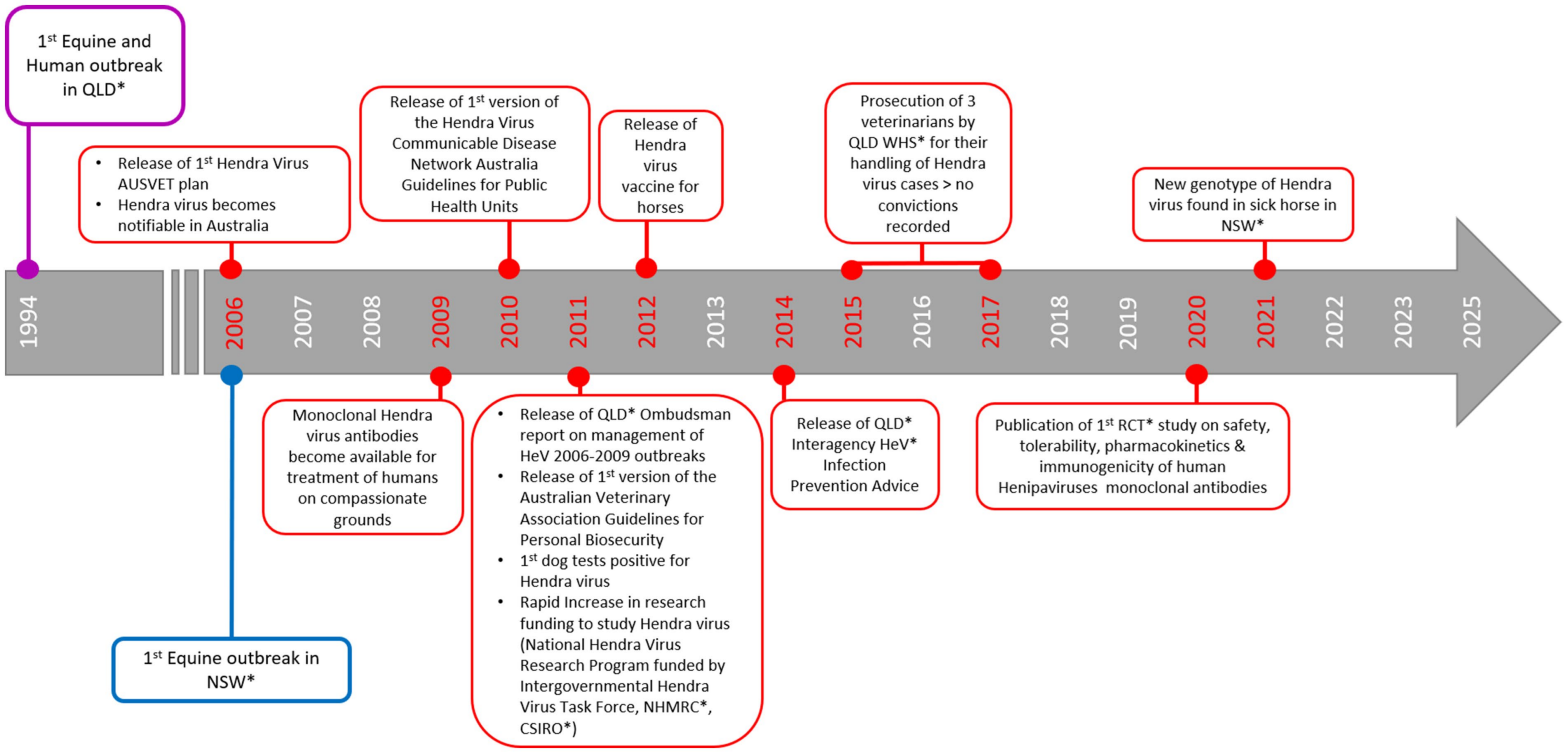
Timeline of Hendra virus outbreaks and key advancements for the management of Hendra virus in Australia. *CSIRO – Commonwealth Scientific and Industrial Research Organisation, HeV – Hendra virus, NHMRC – National Health and medical Research Council, NSW – New South Wales, QLD – Queensland, RCT – Randomised Control Trial, WHS – Workplace Health and Safety (2, 4, 6, 8, 9, 12-14, 18-21, 29).

**Figure 2:**
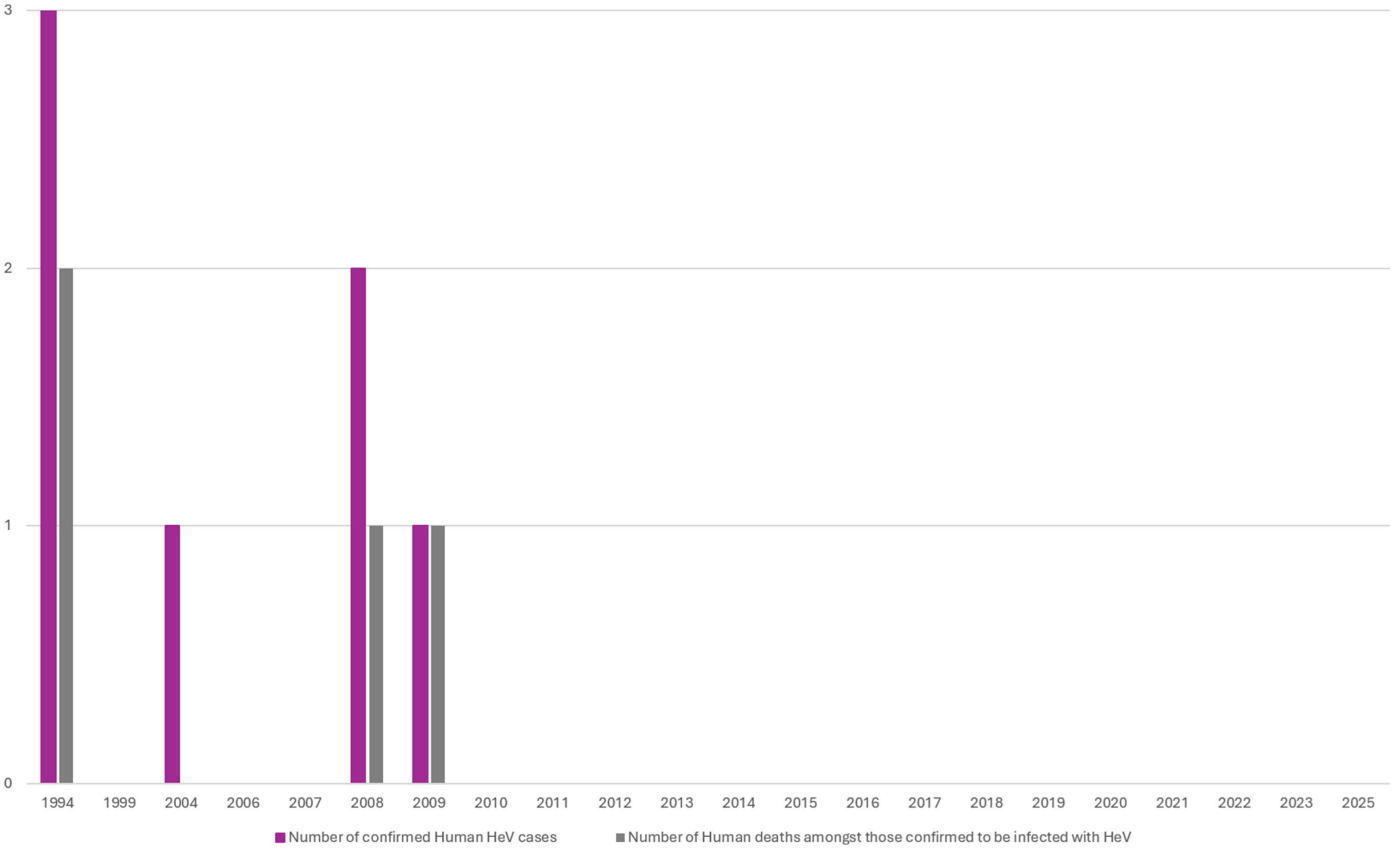
Number of confirmed Hendra virus (HeV) Human Cases between 1994 and 2025 (All cases were recorded in Queensland and represent four fatalities from seven HeV confirmed cases) (6).

**Figure 3:**
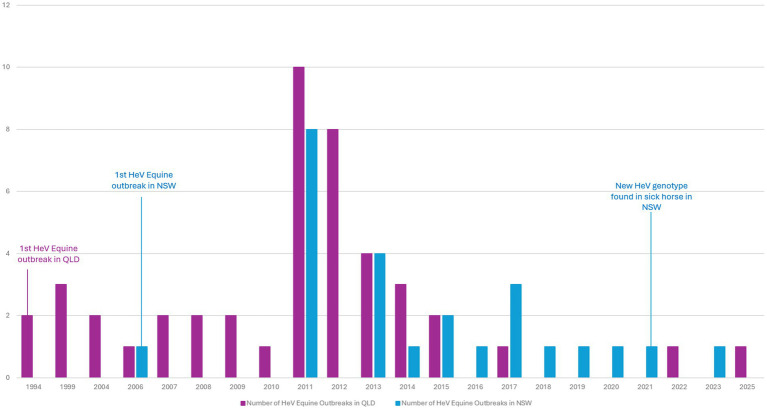
Number of Equine Hendra virus (HeV) Outbreaks per year in Queensland (QLD) and New South Wales (NSW) between 1994 and 2025 (6).

Hendra virus (HeV) was first detected in horses in Australia in 1994 after spill-over infection from flying foxes (*Pteropus* spp.) (1, 2). There have been over 100 equine and four human deaths associated with HeV spillover in the Australian states of Queensland (QLD) and New South Wales (NSW) (3–8) (Figures 1, 2). HeV is a notifiable disease, being considered a Biosecurity Category Level 1 infection and “Emergency Animal Disease (EAD)” (5). Clinical diagnosis of HeV infection in horses remains challenging as signs in infected horses are non-specific, including colic (abdominal pain) and respiratory and/or neurological symptoms (5). Animals with signs of HeV infection frequently die or are euthanised for welfare reasons (9) and strict burial protocols are required (9, 10). While veterinarians and government authorities were initially unprepared to manage HeV (11–13), extensive infection prevention and control guidelines (IPC) were subsequently developed and promoted to safely manage horses and prevent human exposure (11, 12, 14, 15). The figures below show the number of confirmed equine cases (see Figure 1), human cases (see Figure 2), as well as the timeline of HeV emergence in equine and human populations, alongside key developments in HeV management in Australia (Figure 3). One Health recognises interdependencies between human, animal, and environmental health and calls for coordinated action across these sectors (16). In the specific context of HeV, the human–horse bond, veterinary decision-making, biosecurity policy, wildlife ecology, and public communication intersect. In July 2025, a further death of an unvaccinated horse in QLD (4) underscored the continuing need for a coordinated One Health stance across vaccination uptake, rapid joint assessments, and clear public messaging to markedly reduce risk to people and horses.

There is no commercially available human vaccine against HeV (17). In QLD, an experimental monoclonal antibody therapy (m102.4) was provided on compassionate grounds as post-exposure prophylaxis for human contacts considered to have high level exposure (18). A vaccine for horses has been commercially available since 2012 (19, 20). Although vaccination is not compulsory, it can be mandated by equestrian organisations (21). While public perception varies, there is support for veterinarians to decline attendance to sick, potentially HeV-infected horses that are unvaccinated (22). However, this approach may create gaps in care and HeV management, potentially increasing the risk of exposure for horse owners who continue to provide care without veterinary support.

The close emotional bond and affection shared between humans and animals is well recognised (23–25), often leading to significant distress when caring for a loved animal experiencing a deteriorating or terminal condition (26). Feelings of distress in horse owners and veterinarians may be exacerbated when a horse rapidly declines and dies or is euthanised due to HeV infection (27, 28). During responses to the initial HeV cases, particular challenges identified by QLD veterinarians included testing difficulties (11) and communicating IPC and HeV management practices to horse owners (28). Veterinarians also grappled with the complex task of simultaneously protecting themselves, the community, and other horses, while managing the responsibilities of testing, mandatory reporting, and, when necessary, euthanasia (11, 28). Compounding this scenario is the mandated 21 day period of symptom monitoring of human contacts, overseen by public health unit (PHU) staff, during which individuals exposed to HeV are advised to seek medical attention from a general practitioner (GP) or hospital emergency department if they developed fever, respiratory symptoms, or neurological signs (29).

Previous qualitative research has explored horse owners’ attitudes, perceptions, and practices regarding HeV (30), as well as the experiences and challenges faced by veterinarians working in high-risk settings (28, 31). However, little is known about the emotional and experiential factors that shape how individuals respond to the presence of a confirmed HeV case, particularly how they interpret risk, self-protection, and responsibility. Social science methodologies can address this gap (7, 32). This study used Interpretative Phenomenological Analysis (IPA) to explore the lived experiences of horse owners and veterinarians involved in managing horses diagnosed with HeV. Indeed, with over 60% of emerging infectious diseases being zoonotic in nature (33), there is an urgent and growing need for One Health approaches that bridge the gap between human, animal, and environmental health. By understanding the subjective experiences, results have the potential to inform the development of more responsive education, support mechanisms, and intervention strategies that improve outcomes for both human and animal health.

## Materials and methods

### Theoretical framework

Interpretative Phenomenological Analysis (IPA) is both phenomenological and hermeneutic in nature. It is phenomenological in that it seeks to understand how individuals experience and make sense of significant life events from their own perspective. Concomitantly, it is hermeneutic involving a dynamic interaction between the participant’s meaning-making and the researcher’s interpretative lens (34). In this way results extend beyond mere description, allowing for a rich, in-depth exploration of subjective experiences. Importantly, we situated IPA within a One Health frame of reference at three levels—micro (owner/vet–horse interactions, emotions, risk perception, and care decisions during suspected/confirmed HeV), meso (owner, veterinarian and other organisational interfaces, IPC practices, testing, and carcass management), and macro (policy/regulation, media visibility, stigma). This framing guided sampling (owners, veterinarians), the topic guide (risk, care, cross-sector interactions), and analysis (linking themes across levels). Further, IPA provided a valuable framework for investigating complex, emotionally charged, and under-researched phenomena, such as human experiences of HeV disease risk, where individuals must navigate uncertainty, threat, and evolving human–animal relationships.

The biomedical model has previously dominated medical and veterinary discourse, focussing on biological mechanisms and disease pathologies (35). While effective in guiding diagnosis and treatment, this reductionist approach often overlooks the complex, lived experiences of individuals navigating zoonotic threats in real-world settings. In contrast, the One Health approach (36) offers a systems-oriented, transdisciplinary framework that recognises the interdependence of human, animal, and environmental health.

Semi-structured interviews were conducted and analysed using IPA (37). Horse owners/guardians (hereafter referred to as ‘owners’) and veterinarians were eligible for inclusion if they were confirmed contacts of equine HeV cases in northern New South Wales at the time of an incident (collected under the NSW Public Health Act 2010). Of the 29 owners initially contacted, 15 were reachable. Six declined to participate, while nine agreed to receive study information via email and were given the opportunity to ask questions. Of the 13 identified veterinarians, five were successfully contacted and agreed to receive study information. A total of eight owners and five veterinarians provided written informed consent and were subsequently contacted by the primary author to schedule interviews. Participant recruitment took place in April and May 2024. Approval for this project was obtained from the Hunter New England Health Human Research Ethics Committee (Ref: 2023/ETH02947).

### Data collection

Four semi-structured interviews with owners were conducted face-to-face on affected properties and four were conducted via video conferencing due to participant preference or relocation to another setting. One veterinarian was interviewed in person and four were interviewed via video conferencing. Basic demographic and background information was elicited at the start of the interview. In line with recommendations by Smith et al. (37) the topic guide (see Table 1) was developed and operationalised flexibly in structure with open ended questions designed to be non-directive and participant-led. This enabled participants to focus on their exposure experience, voice any concerns of their choosing, and share lived experiences in the context of biosecurity risks. There was no financial recompense for participation in the study.

**Table 1:** Participant interview guide.

Purpose	Questions	Probes
Warmup questions	Tell me how you got into horses/being a vet?	
Asking for permission	Can we talk about the Hendra case that you experienced?	
Focusing the interview	(For owner/carer only) Can you please tell me about the horse?	What was he/she called? Whose horse, was it? How long had it lived here?
The event	Can you please take me back to when you first became aware that the horse was sick? And then what happened? Continue until the participant feels they have reached ‘the end’ of the event for them.	Probes for chronology, e.g.: what happened then/before that/at the same time? Probes for narrative depth: who, what, where, when and why? Phenomenological probes, e.g.: What was that like for you? What did you think that meant? How did you make sense of that then? Looking back, how do you make sense of that now?
	Can you please tell me what public health measures were advised?	Phenomenological probes, e.g.: What was that like for you? What made it easy or difficult? What would help in the future?
Personal reflections of enablers	What helped support you at the time?	Interviewer may give their summary of the event and use key moments to ask about enablers to following vet advice/protocols.
Personal reflections of barriers	What made the situation more difficult for you at the time?	Interviewer may give their summary of the event and use key moments to ask about barriers to following vet advice/protocols.
Takeaways	If you had to face this situation again, what would you do the same?	What would you do differently?
Helping others	What would be your advice to (other) horse owners/vets?	Vaccination.

### Data analysis

Interviews were audio recorded and transcribed verbatim by an external transcription service, with identifying data removed. Transcripts were checked for accuracy by the primary author (JW) and analysed using an IPA approach (37). To preserve role-specific meaning structures, we first analysed horse owners and veterinarians separately using identical procedures, then conducted a comparative synthesis to map convergences and divergences. Two authors (JW, social scientist; DVB, clinical nurse consultant) undertook idiographic, line-by-line analysis of each transcript—moving between parts and the whole in the hermeneutic circle—with descriptive, linguistic and conceptual commenting (37). For each case, JW and DVB drafted experiential statements and tentative themes, grounded in verbatim quotes and case summaries. Only after completing within-case interpretation did we examine patterns across cases, attending to convergence and divergence to elaborate superordinate themes. JW and DVB met iteratively to interrogate and refine interpretations, repeatedly returning to data to test developing claims. The resulting superordinate themes and sub-themes were first shared with DD and RF for peer debriefing, then with the full authorship team (spanning clinical and academic backgrounds) for critical dialogue and refinement. Superordinate and related sub-themes were revised over several iterations until consensus was reached, with decisions documented in an audit trail. We distinguished superordinate themes as higher-order, recurrent patterns attributed by horse owners and veterinarians across cases, and sub-themes as more specific, theoretically coherent facets nested within a given superordinate theme. These were developed iteratively by checking against data extracts and the identified concepts. We adopted key strategies to enhance study rigour. Dependability and the ability to replicate this study was enhanced using an audit trail, a clear analytic process and peer debriefing (38). Credibility and confirmability was achieved through reflexivity, transparency in thorough team discussion, and the use of verbatim quotes (38).

## Findings

### Participants

Most owners were female (*n* = 5) and owned between 1 and 12 horses (average = 3). The human-horse relationship and reasons for ownership varied; participants owned horses for competition (racing and show jumping), companionship, child ponies, as caretakers of retired competition horses, or to prevent horses (particularly retired racehorses) from being sent to slaughter. Some owners also housed horses for others. Of the veterinarians interviewed, four were male and one female, representing the NSW Department of Primary Industries (DPI), the government agency Local Land Services (LLS), or private practice. The DPI is responsible for agriculture, fisheries, aquaculture, forestry, and biosecurity, while LLS is a NSW government agency focused on biosecurity emergencies and natural disasters. Demographic data is limited to protect participant anonymity.

We identified four superordinate themes for owners, and three superordinate themes for veterinarians; a brief comparative synthesis follows. Exemplar quotations are provided to evidence each theme and show coverage across cases and their corresponding year. Owner participants are noted as ‘P’ and Veterinarian participants are noted as ‘V’.

## Horse owners

### Theme 1: “What is going on?”: cumulating stress when responding to a horse with HeV

#### Responding to a sick horse

Owners were experienced and quickly recognised when their horses appeared sick or were acting out of character. Owners reported closely monitoring their horses, with some contacting the veterinarian during the night when they did not think a deteriorating clinical situation could wait until the morning. Others called the veterinarian upon discovering a deceased horse upon waking.

He just did not look himself. I am thinking, “What is going on with you?” Because physically he looked fine, other than just a little bit depressed looking. It did not make sense, so we ended up calling the vet and having the vet come out. (P6, 2016).

#### Swab testing difficulties

All owners reported that the attending veterinarian suspected something serious and took precautions including donning personal protective equipment (PPE) and taking a swab to test for HeV. Several owners noted that obtaining a swab was challenging, particularly when horses were distressed by the unfamiliar, cumbersome, and noisy appearance of veterinarians in PPE. In some cases, veterinarians struggled to get close enough to a frightened horse to collect a sample. One owner assisted the veterinarian in obtaining a swab by catching a horse that was healthy and isolated from the infected animal for a vet; the owner was not wearing PPE.

And it was very hard to get them (swabs) when we were dressed in all that (PPE). (The vet) got all of them except one and said to me, “you are just going to have to take the suit off (PPE) and get [catch] him.” (P2, 2014).

#### Caring for a deteriorating horse

Two owners expressed confusion and anguish when they observed their horses suffering before they died. They struggled with how to provide care and risked exposure while continuing to offer comfort, such as providing food and water, and covering the horses with blankets to keep them warm.

I thought like, well there is nothing else I can do. I have got to do this for the horse because he cannot feed himself. I have already been in contact with him anyway, so if I am going to get it (HeV), you know, I would have it… But I was making sure to wash my hands thoroughly. (P6, 2016).

### Theme 2: “absolutely inundated”: the experience of grief when overwhelmed by the involvement of external agencies and anxiety compounded by community stigma

#### External agencies involvement

In response to HeV being a notifiable disease, owners reported they were “absolutely inundated” (P4, 2014) and overwhelmed by the involvement of multiple agencies, including DPI veterinarians, LLS staff, the PHU and research organisations appraising flying fox ecology and environmental issues. Despite the overwhelming nature of this involvement, owners expressed gratitude for the guidance they received, particularly in reducing ongoing biosecurity risks and being included in the burial process.

(The vet) got suited up and said (to me) look, technically you are not supposed to be here (for the burial), but if you stay 100 yards up the hill that will be okay. (The vet) made that all as painless as possible. (P2, 2014)

While owners were aware of the risk involved, many took the opportunity to say goodbye to their horse. Indeed, owners’ responses suggested they would touch a horse, despite being advised not to, to provide care, say goodbye and to give and receive comfort. However, all participants reported wearing PPE during these interactions.

We (knew we) could not touch her really. Oh, we did. But we all had to be suited up in all the PPE gear and stuff, yeah…. (P1, 2011).

#### Experience of stigma

Owners reported that the involvement of broadcast media exacerbated their feelings of distress. While most acknowledged that DPI veterinarians made efforts to *“protect their identity for as long as they could”* (P4, 2017), this was not always practical depending on the location. For example, the placement of biosecurity signage on property fences or attempts by local media to discover and report the exact location of HeV cases made anonymity difficult to maintain.

Some owners experienced negative backlash and stigmatisation from the community when knowledge that their horse had HeV infection became known, typically in response to media reports. Perceived stigma was heightened among owners of early HeV horse cases when little was known about HeV. In contrast, one owner chose to proactively inform the local community due to their proximity to a racecourse and recent involvement in camp mustering. Another owner recalled a compassionate community response, noting that “*they felt very sorry that I lost two really good horses*.” (P3, 2011).

Owners who had been exposed to an HeV infected horse or who were receiving human monoclonal antibodies were provided with information and advised to contact their GP if they developed symptoms. However, owners experienced further anxiety and stigma when the GP practice reacted with shock and concern and implemented transmission based precautions, such as the use of PPE.

So, we went home (with a letter to give to our GP of a record of what’s been done). Well, that turned into a bit of an education as well. I walked into the doctor’s …. they had us out of there straightaway, masks and gowns and out in the backrooms by ourselves. (P2, 2014).

### Theme 3: “they were contacting me every day”: the experience of public health unit support for personal risk

#### Personal risk

All owners reported that the PHU conducted a formal risk assessment of their personal and other contacts’ level of exposure. For some, this was the moment when the reality of personal risk became clear. Urgent referrals to receive human monoclonal antibodies further heightened their perception of risk, particularly when children were involved.

So, the tragedy is the horse, (was) quickly overshadowed by the children - actually, the kids having to have monoclonal treatment. (P5, 2017).

Most owners reported feeling *“well supported”* (P6, 2016) by phone contact from the PHU. Feelings of support were strengthened when owners perceived that other people they cared about, who had been in close contact with their horse, were also being shown concern and assessed for risk of exposure.

#### Mode of support

All owners reported feelings of grief and shock which were exacerbated by fatigue due to lack of sleep, worry, and waiting for test results for their horse or themselves. When asked if a personal visit from the PHU to provide education, advice, and support would have been preferable to phone calls, responses were mixed. Some owners felt that an in-person visit would have been *“overwhelming for me”* (P6, 2016) and *“just been somebody else whom you had to deal with at the time”* (P2, 2014).

I still probably would have sat there in shock and not talked. (P3).

### Theme 4: “PPE is not practical”: caring for a sick horse trumps biosecurity measures and is compounded by vaccine concerns

#### Implementing infection control procedures or not

Owners reported the DPI provided advice on care for other horses who were considered at risk of infection. A few owners also reported that their dogs were isolated and monitored. The threat of losing other animals, particularly pet dogs, was a further source of distress and anguish, especially for children.

The horse being sick was one thing, but someone threatening to put your dogs down, that was a whole other level for them (children). So that was their (children’s) plan to protect the dogs–to run away (with the dogs). (P4, 2017).

#### IPC challenges

Most owners reported having their horses vaccinated once the HeV vaccine became available and they continued to do so. However, some expressed vaccine hesitancy, choosing to refuse or discontinue vaccination *“due to stories you hear*” (P8, 2015). Over time (since the initial awareness of HeV), it appeared that vaccination was not at the forefront of owners’ minds. Some stated that they were *“not sure”* (P5, 2017) about the vaccination status of their horses, particularly newly acquired horses or those being housed on behalf of others.

Owners reported they never used PPE to avoid distressing their horse and were unlikely to use PPE if faced with further high-risk events. Overall, PPE was considered impractical or unsuitable due to the horses’ sensitivity and reactivity. As a result, some owners emphasised the need for a pragmatic and practical approach to care, while others advocated for vaccination as the most effective way to mitigate risk.

PPE, well it’s not practical for starters. And my horses are pretty forgiving compared to most. They do not like it (PPE) so it’s going to make everything you do difficult. I just think like, just get the bloody vaccine and you do not have to worry about it. (P2, 2014).

## Veterinarians

### Theme 1: “what am I doing this job for?”: job challenges and risks taken when responding to high consequence infectious zoonotic disease

#### Impact on work practices

The emergence of HeV had significant implications for both government and private veterinarians, particularly their work practices and the risk of exposure for themselves or their staff when attending to sick, unvaccinated horses. Some veterinarians reported experiencing backlash from the community, with perceptions that promoting horse vaccination was a money-making exercise and that they lacked concern for the welfare of sick animals. This contrasted with the ethical dilemma veterinarians faced in balancing workplace health and safety responsibilities with their professional duty to provide care for sick animals.

HeV was the worst thing that happened to us vets on the frontline. We were hung out to dry with impossible situations. The horse population thought we were just trying to make a dollar out of vaccinations …. yet everywhere we looked there was no safe way of doing this. You cannot send an employee to see a horse that is unvaccinated…. but horses that are very very sick are in very very great need of a vet. (V5, 2013).

All veterinarians reportedly approached potential cases with caution, including the implementation of IPC protocols and careful planning to prioritise treatment while awaiting results of suspected HeV cases. There was notable anxiety when deciding whether to attend sick, unvaccinated horses. Indeed, a positive case often involved deep reflection, as veterinarians critically evaluated their processes to reassure themselves that they were *“reasonably confident that everyone (staff and owners) were safe.”* (V4, 2011/13). Even waiting for laboratory results had implications for decision making and the care provided to the sick horse.

We are only going to give minimal treatments - and that potentially could mean that the horse is missing treatment for something that could be easily treated (while we are waiting for the sample to be returned). (V4, 2011/13).

Some veterinarians reported feeling stressed when managing owners’ responses while assessing a dynamic and evolving situation. The complexity of these scenarios often involved attending to a very sick and distressed horse while simultaneously ensuring the safety of owners, especially when they were contesting the advice being provided.

I was unable to get the owners to back away from him (horse), they would not. Eventually I said to (one of the owners) you actually have to leave the paddock. (V2, 2013).

#### IPC challenges

Veterinarians reported that adhering to IPC requirements, particularly donning and wearing PPE was time-consuming, making it difficult to, *“get through a day’s work”* (V4, 2011/13). In addition to the practical difficulties, PPE also caused unease and personal discomfort. A key aspect of clinical decision-making was determining when PPE was necessary, especially in situations where the risk was not immediately visible or recognisable to the horse owner. These challenges were further amplified when dealing with difficult field situations, outside of a controlled clinical environment.

There was a lot of resistance too because it (PPE) is uncomfortable and hot and sweaty, it takes 10 min to put this thing on if you do it properly. It is often boiling hot, and they (owners) just cannot see the point, especially when most of the time it is going to come back negative. (V1, 2011).

Facilitating horse burial while wearing full PPE was hot and physically demanding, with one veterinarian reporting they came close to “passing out” (V1, 2011) while supporting the excavator operator and the horse owner.

#### Personal exposure

As with owners, veterinarians who were considered at high risk of exposure were advised to seek medical advice if they developed symptoms. Common feelings of stress, uncertainty, and isolation were amplified during the influenza season, as influenza symptoms mirror those of early HeV illness in humans. Additionally, navigating follow-up care after initial contact with the PHU was less streamlined in earlier years, with inadequate communication of risk levels and poorly established processes. Veterinary participants highlighted the need for more support and reassurance when consulting the PHU about symptoms of concern following HeV exposure. Although processes improved over time, the anxiety experienced underscored gaps in communication.

And I was told by the PHU that if I had flu-like symptoms or headaches that I should seek medical advice. And it happened to be flu season, so I was a bit stressed. But then, that was it. I did not hear from anybody ever again, that was the last I ever heard of it. (V3, 2013).

### Theme 2: “you have to be familiar and sensitive to people”—dealing with clients in distress

#### Dealing with clients in distress

All veterinarians reported being used to dealing with challenging scenarios, including undertaking risk assessments and supporting clients in distress. However, managing a high-consequence emerging infectious disease (3) that required explaining to and adequately supporting clients was time-consuming, with one veterinarian noting it “*took a couple of hours… (and) you just let them talk themselves out*” (V1, 2011). This was often coupled with heightened stress, which was further exacerbated by the need to limit the risk to other animals.

Yeah, a lot of dealing with the owners. In one of the cases I managed a dog, tested positive. That was obviously a bit challenging telling the owner that we were going to euthanise their dog. (V3, 2011).

Stress was compounded when the owner resisted advice regarding the seriousness of the HeV risk and did not recognise the veterinarian’s authority.

I was trying to be gentle and kind and compassionate about their misunderstanding about what was happening. I really needed them to focus and yeah it was really tough… (V2, 2013).

#### Stringent measures needed to prevent further exposure

Since owners were perceived to be in *“an emotional state”* (V2, 2013) some veterinary participants reported uncertainty about the extent to which they complied with IPC protocols. However, veterinarians noted that owners were more likely to follow advice once HeV was confirmed in their horse.

Once you have got a diagnosis, I think people are generally a bit better at following your advice. It’s probably those cases where you have a horse which is a suspect, and you do not yet have the lab confirmation - they are the ones that do not necessarily take the advice. (V3, 2011).

Veterinarians expressed concerns about the professional and legal *“consequences for not handling things appropriately”* (V4, 2011/13). For example, one veterinarian required the “client or owner of the horse to sign a declaration acknowledging that samples had been taken for possible Hendra and all the associated risks” (V4, 2011/13). Concern was heightened when it was perceived that horse owners had placed themselves at risk.

Preparing for horse burial was complex. While burials were conducted as soon as possible after euthanasia or death, veterinarians had to advise already distressed owners not to approach or touch their beloved animals, instead covering them with a tarpaulin while waiting for test results. Participant reports highlighted the challenges of balancing compassion with education while adhering to burial protocols.

Often times they are a really beloved pet, and it has died….it is pretty devastating. And then you are saying to them also, “You are not allowed to bury it and do not go near it.” They get pretty upset by that. (V1, 2011).

#### Extent of support needed

Given the fatigue and distress experienced by owners following the death of a horse and the risk of HeV, one DPI veterinarian emphasised the importance of the PHU conducting face-to-face risk assessments. This participant perceived site visits were seen as more effective in providing support to horse owners and obtaining accurate information regarding exposure, compared to phone call assessments. While face-to-face follow-up by the PHU was contrary to horse owners’ reported preference, one veterinarian noted that owners “*just clam up [over the phone to PHU] and do not give the full information - like they would just in a conversation that is one on one (face-to-face)”* (V1, 2011).

## Discussion

### Key findings

This study offers the first in-depth exploration of the emotional and behavioural responses of horse owners and veterinarians to a rare but high-consequence Hendra virus (HeV) event. Participants reported prolonged uncertainty and emotional burden at the horse–human interface; operational barriers to safe attendance (PPE, time, carcass management); and variable cross-agency coordination. Within a One Health framework, we recommend that decision making support is provided to owners regarding horse vaccination and on-property biosecurity; optimising veterinary safety through adequate resourcing and access to standardised joint Standard Operating Procedures (SOPs) for sampling and carcass/burial. We also posit the need for psychosocial support for owners and responders. In particular, the findings point to the central role of the, owner–horse bond in shaping decision-making and risk perception and highlight the ethical and legal tensions experienced by veterinarians in balancing duty of care to animals, clients, and public health. These insights underscore that effective HeV risk management requires One Health-aligned responses that address emotional and relational dynamics alongside regulatory measures.

Our findings mirror prior outbreak literature. In Ebola responses, support for safe and dignified burials improved adherence to protocols when communication was coordinated, community-engaged, and grounded in trust (39). Our results also parallel veterinary experiences during foot-and-mouth disease in the UK and African horse sickness in Thailand, where the need for explicit burial/carcass-disposal protocols and isolation was emphasised (40, 41). Likewise, we underscore the importance of clear carcass-disposal SOPs, movement controls, and coordinated mental health support in addition to open discussion of the benefits of vaccination to reduce mortality (42) and improve outcomes for people and animals during suspected or confirmed Hendra virus (HeV) events.

Developments in human and animal physiology and psychology outline the positive emotions shared by both humans and horses in a mutually affective relationship (43). This bond has been shown to improve coping, resilience, and reduce stress (24, 44). The depth of the relationship is further emphasised by evidence that some horse owners/carers are willing to risk their lives for their horses, as seen during bushfires (45) or accidents (46). Such responses help explain why it is difficult for horse owners to adhere to recommendations to avoid physical contact with a distressed and suffering horse (47). Similarly, the psychological distress experienced by veterinarians has been well documented and is magnified when facing moral and ethically and legally challenging clinical scenarios (48). These findings underscore the importance of a One Health approach (20) which emphasises collaboration, communication and cooperation between PHUs, veterinarians (private and DPI) and healthcare providers. We advocate for enhanced professional education for veterinarians and agricultural personnel—emphasising risk communication, ethical decision-making, and grief/compassion-fatigue management. Proactive referral to mental-health support for those directly involved in serious zoonotic events is strongly recommended, given well-documented psychological impacts in animal-health emergencies and established guidance on Risk Communication and Community Engagement (RCCE) and veterinary wellbeing (42, 49–51). Furthermore, our findings suggest the need for PHUs to take a more active role in supporting GPs when their patients have been exposed to zoonotic diseases. Relying on affected individuals to inform or educate their healthcare providers risks reinforcing feelings of isolation, increasing stigma, and compounding emotional distress for both horse owners and veterinarians. Ensuring that GPs receive timely, evidence-based guidance directly from public health authorities would support a more coordinated and compassionate response.

### Practical implications

Consistent with past research, some veterinary directives were readily followed by horse owners (52, 53). However, veterinary advice was perceived as obsolete when owner exposure to HeV had most likely already occurred by the time a veterinarian attended (11). Such findings may reflect the historical absence of clear risk mitigation guidelines when HeV first emerged (13, 54) and difficulties communicating with horse owners about HeV risk of exposure and IPC measures (28, 55). Central to this study were challenges experienced by owners and veterinarians when adopting IPC, especially the use of cumbersome and uncomfortable PPE around horses in less-than-ideal environmental conditions. In some cases, the use of PPE was deemed unsafe around horses, posing an occupational health and safety dilemma. Echoing the experience of QLD veterinarians (11), our findings confirm the real-life demands of working in non-clinical settings in full PPE (56). Results underscore the negative experience of using PPE during HeV response as a critical factor that must not be underestimated. Preparation for future outbreaks should incorporate recommendations that extend beyond technical protocols to include cost considerations, practical usability, and clear delineation of responsibilities (11, 28). For instance, improved PPE design may enhance both uptake and compliance in high-stress situations. Additionally, subsidies or financial support for safe first-responder private veterinarians who suspect HeV infection are warranted to ensure there is adequate time and resources to ensure thorough and safe investigation. First responder and PHU staff may benefit from education on risk communication approaches that acknowledge the impact of the owner–horse bond. Concerning the management of those exposed to HeV, we posit the need for communication between the PHU and GPs about symptom management as well as integrated mental-health referral pathways for owners and veterinarians.

Although current IPC guidelines for HeV response are clearer than in the past, the emotional toll experienced during an outbreak has not been explicitly addressed, nor has targeted support been provided to horse owners and veterinarians. Veterinarians, as likely first responders, face a complex role, balancing the need to manage owner distress with their responsibilities related to biosecurity, public health, and workplace health and safety, while also delivering support and safety education. However, how horse owners respond to veterinary directives is shaped by personal views and lived experiences, including vaccine hesitancy, complacency, and cost-related concerns. We draw attention to the potential benefit of tailored harm reduction interventions that incorporate emotional preparedness—approaches that are pragmatic, compassionate, and non-judgemental, as advocated in other harm reduction research (57). We hypothesise that emotionally prepared horse owners may be better equipped to comprehend, process, and act on veterinary advice during a HeV event. Framed within a harm reduction model, inviting owners to reflect in advance on the potential impact of a HeV outbreak on their horse, family, community, and other animals may increase openness to preventative actions, including vaccination.

### Strengths and limitations

A key strength of this study lies in its concurrent exploration of the real-world experiences of both horse owners and veterinarians in response to a confirmed HeV case. Consistent with the principles of IPA, the study enabled an in-depth understanding of how individuals make sense of emotionally complex and ethically challenging events, based on a small but information-rich sample. However, several limitations are acknowledged. Participants with strong views or emotional experiences, may have been more likely to respond. In addition, there is a risk of recall bias, as participants were reflecting on past events that may have occurred at different points along the timeline of HeV awareness and IPC protocol evolution. Consequently, contextual differences (changes in public health messaging, availability of PPE, and clarity of guidelines) may have influenced individual experiences and perceptions. Despite these limitations, the study offers novel insights into the lived experience of zoonotic disease response that can inform future policy, education, and supportive strategies.

## Conclusion

Positioning lived experience within a One Health operating model clarifies where shared actions (communication, SOPs) and role-specific supports (veterinary resourcing, owner psychosocial care) can reduce risk and improve response during suspected or confirmed HeV events. A central finding was the profound grief and emotional strain experienced by participants, arising from perceived personal risk, concern for loved ones and other animals, and the longer-term burden of managing the consequences of exposure. The findings highlight the need to broaden the role of PHUs to include recommendations for, and facilitation of, appropriate mental health support for those directly affected by zoonotic outbreaks. In parallel, further research should focus on developing targeted, emotionally sensitive public health messaging tailored to the needs of animal owners and veterinarians. Such efforts are essential to improving the overall response to zoonotic outbreaks, ensuring that both physical and psychological wellbeing are adequately addressed in One Health frameworks.

## Data Availability

The raw data supporting the conclusions of this article will be made available by the authors, without undue reservation if ethical and data security requirements are met.
